# Examination of social media nutrition information related to multiple sclerosis: a cross-sectional social network analysis

**DOI:** 10.1017/S1368980025100943

**Published:** 2025-09-18

**Authors:** Yasmine Probst, Emiliana Saffioti, Sarah Manche, Melissa Eaton

**Affiliations:** School of Medical, Indigenous and Health Sciences, University of Wollongong, Northfields Avenue, Wollongong, NSW 2522, Australia

**Keywords:** Multiple sclerosis, Nutrition information, Social media, Online

## Abstract

**Objective:**

Multiple sclerosis (MS) is a chronic, auto-immune, neurodegenerative condition with increasing global prevalence. People living with MS (plwMS) have reported limited guidance relating to nutrition information. Paired with varied health literacy levels, this makes plwMS susceptible to nutrition misinformation.

**Design:**

A cross-sectional online social network analysis (SNA) examining nutrition information for MS.

**Setting:**

A systematic SNA using Twitter/X and YouTube platforms using NodeXL to summarise metrics. Quality was assessed using the QUEST tool. Content analysis of YouTube videos was synthesised into themes for misinformation.

**Participants:**

Online publicly available social media user posts and video content.

**Results:**

Twitter/X SNA revealed keywords used most by an account representing 72·8 % of the user network with common diet mentions including Wahls (57 times), paleo (15 times) and ketogenic (11 times). ‘Favourite count’ metrics were strongly correlated with ‘repost count’ (r = 0·83, *P* = 0·000). Videos which endorsed a diet were more likely to have a lower QUEST score. User engagement metrics were higher for lower quality videos. The quality of online nutrition information relating to MS was moderate (61 %). Physicians were the most likely source of nutrition information endorsing a diet for MS. The content analysis identified a knowledge gap for both medical professionals and plwMS.

**Conclusions:**

Nutrition misinformation for MS occurs on social media and information quality is variable. Audiences need to be cautioned about users with large followings and evaluate the credibility of all information. This study reiterates the importance of evidence-based information for the MS community.

The Internet is largely participatory, focused on sites and services that rely upon user-generated content including social media^(1)^. To date, social media is one of the most popular online activities and has been instrumental in expanding the communication of health messages to a diverse audience. The social media audience is predicted to reach near 6 billion consumers by 2027^(2)^ with almost 80 % of countries now using social media as a primary means of health information; searches for health topics are the third-most popular online activity^(3)^.

The Internet has facilitated an extensive network of user-generated content on a range of topics, playing a role in the transmission of health information and a means to facilitate the transfer of data between content producers and consumers^(4)^. However user-generated data often lack author credibility which may facilitate information that is of poor scientific quality^(5)^. The Internet may also limit user access to health information for specific population groups creating access inequalities to health information^(6)^. These challenges have led to questions around the accuracy, validity, and comprehensibility of online information^(7)^.

Accuracy becomes increasingly important when health-seeking behaviours are from consumers with chronic conditions. Multiple sclerosis (MS) is a chronic, progressive, neurodegenerative condition characterised by autoimmune demyelination of the central nervous system^(8)^. Health behaviour (lifestyle) management can slow disease progression and improve quality of life^(9,10)^, though first-line therapy is pharmacological. Changing nutrition or eating behaviours for disease management of MS is supported by limited and inconsistent evidence^(11)^. No specific dietary recommendations exist, and people living with MS (plwMS) are advised to follow national dietary guidelines. The lack of specific guidance leaves individuals newly diagnosed with MS vulnerable to nutrition misinformation as they attempt to self-manage their condition^(9)^. This also creates an opportunity for ‘diets’ to be promoted that exclude whole food groups or food components. Restrictive approaches to eating, in the long term, can result in nutritional inadequacy if not monitored by a healthcare professional. Common ‘diet types’ focused on MS include the low fat/saturated fat (Swank), paleolithic or modified paleolithic (Wahls), plant-based (Overcoming MS, McDougall), gluten-free and/or ketogenic approaches often promoted as components of lifestyle programmes and not always evidence-based. While small-scale studies have shown change to MS-related fatigue and quality of life^(9,10,12)^, large and high-quality studies are lacking.

A recent study found that 82 % of plwMS reported gathering medical information online before their first healthcare appointment^(13)^. As noted earlier, social media forms a large portion of this online information and is available to the consumer in an unregulated space. With users spending more than 2 hours per day on social media, an examination of the information is vital as plwMS have previously described that there is ‘*both too much information online and too little that applied to them*’^(2,14)^.

An online social network analysis (SNA) is an assessment that permits exploration of information networks through the synthesis of digital traces of human behaviour^(15,16)^. When combined with health information-seeking behaviours, the networks may be considered to be learning networks according to connectivist learning theory concepts which emphasise the role of connections in the process of learning and teaching^(17)^. It must be acknowledged that the impact of this learning is affected by the user, particularly for vulnerable populations^(18)^. To date, no studies have reported online SNA for the MS community. Therefore, this study examined nutrition information related to MS available via social media platforms as participatory Internet sources.

## Methods

This cross-sectional study adapted methods of SNA from previously published studies^(19–21)^. Social media platforms with active public use and data availability, namely Twitter/X, due to its indexing and reach, and YouTube, as the second most used social media platform, reaching over 2500 million users in January 2023, were examined in our study^(22,23)^. While it is recognised that originally Twitter/X was for professional use, it shifted as a platform for health-related information^(24)^ where consumers raise awareness, share experiences, and combat stigma^(25)^. Of note, this study was conducted before Twitter was rebranded to X and, therefore, will be referred to as Twitter/X for consistency. Facebook and Instagram platforms were excluded due to limitations of privacy and data availability as posts are often housed within specific community groups or required personal connections. At the time of our study, human research ethics guidelines were not available via the Ethical Code of Conduct used in Australia. We have, however, maintained ethical principles in the data that we share. No direct quotes were used from Twitter/X or the transcripts from YouTube. Consent was not obtained directly from the authors of either platform as the data were publicly shared to very high numbers of users. Under the terms and conditions of the platforms, as researchers, we maintained the user voice and intent when posting their data. We did, however, aim to maintain privacy by ensuring that the use of names in the profiles/handles were replaced with pseudonyms. Where some programmes were named after an individual, we have taken care to only share data related to the scope of the profile/handle, for example, number of followers, likes/dislikes, and comments.

### Data retrieval and extraction

Longitudinal searches were run with both the Twitter/X and YouTube platforms across a 1-week period (June 2021) using NodeXL software (Social Media Research Foundation, 2016) for SNA^(26)^. The Twitter search application interface, as it was known before the rebranding, was used to systematically retrieve the data. The 1-week period captured weekday and weekend variation and was selected due to the growing global and daily usage of social media^(2)^ with no events of public interest related to nutrition occurring in June^(21)^.

Keywords were established through pilot searches and revealed that word networks related to the words ‘multiple sclerosis nutrition’ and ‘multiple sclerosis diet’ within both platforms (Twitter/X and YouTube) were limited in their size, that is, number of connections between users. The networks within SNA are based on connections between elements, for example, words and users, as was explored in our study. As there are limited studies exploring MS health information using social media, the final approach to data retrieval for Twitter/X was developed in consultation with the NodeXL developer.

An MS community user network was created for our study to increase the data availability. A user network is a group of social media accounts with a common theme. The method for creating the network was informed by the dietitian network created in our previous study^(19)^. To balance the network size with the relevance of data, our MS community user network for Twitter/X was informed by the top ten users based on follower number identified from the YouTube network. As user networks are generally larger than word networks, a text analysis was also performed to include only posts that included the terms ‘nutrition and diet’. Users were not restricted by country though we did restrict the language to English language posts.

A separate YouTube search was performed using the same keywords as for Twitter/X, allowing all video details to be considered for subsequent content analysis. We created a new YouTube account to limit the personalisation of the search results. All video outputs were viewed. Videos were sorted using the relevance function to reflect the information that people diagnosed with MS would obtain when searching online. Videos not recorded in the English language or that did not discuss diet and MS were excluded manually. Due to the limited number of items related to MS, videos were not limited by country or by user. Where a user posted more than one video, each video was considered independently in the data analysis. Media ‘type’ was restricted to video format only.

Video titles and Uniform Resource Locators (URL) were exported to Microsoft Excel (Microsoft Corporation, USA, version 16.30). Extracted data were based around the connectivist learning theory and its relevance to online information-seeking behaviours^(17)^. Data extracted from the videos included type of video (e.g. interview/webinar), date posted, video length, view count, number and percent of likes/dislikes (as a percentage of view count), number of comments, source name and category, type of healthcare professional (if applicable), number of subscribers, dietary approach endorsed including national dietary guidelines, country of origin, and MS prevention/management/both. The following extracted data were further dichotomised into Yes/No options for analysis: YouTube account verification for viewer reassurance, original video/repost, endorsing a ‘diet’ for MS, cited academic references and option for viewers to purchase related content.

### Data analysis

#### Social network analysis

A multi-stage, semi-automated process of SNA was employed. Posts and YouTube videos were imported to NodeXL (SMR foundation), with open search filters, with an *a priori* rate limit of 1000 posts and 100 YouTube videos, respectively^(26)^. Duplicates were removed automatically. NodeXL calculates various metrics from the data and creates visuals of the patterns and connections which can be explored, as outlined by Smith et al.^(27)^ The SNA metrics obtained from NodeXL included vertices (users), edges (relationships), cluster algorithms (groupings), graph visualisation and centrality measures. The meaning of each metric is provided in Table 1. The visualisations of the networks, or how the users interact, was clustered using the Clauset–Newman–Moore algorithm which considers groups of densely related posts that share common properties such as tags and descriptors^(28)^. The configuration of the network was identified based on methods outlined by Smith et al.^(29)^

**Table 1.** Definitions related to social network analysis metrics

Metric	Definition
Vertices	Also known as nodes, users, entities or people. The number of users is the number of people or things (e.g. videos, posts) within the network.
Edges	Also known as ties, relationships, connections or links. Edges represent the connections between users.
Cluster algorithms	Algorithms which identify and group network users that possess similar characteristics.
Graph visualisation	A graph representing the relationships amongst users in the network.
Centrality measures	Determine the importance or prominence of a user within the network.
Betweenness	The users at the centre of the network (most influential users).
Degree	The number of connections linked to a user.
In-degree	The number of connections that point towards a user).
Out-degree	The number of connections that the user points to.
Eigenvector	More sophisticated measure of centrality that allows for connections to have a variable value (connecting to some users has more benefit than connecting to others)

The PageRank metric, or an indicator of the influence of one user within a network, was computed to create a ranking of the Twitter/X handles (users) based on their location in the network structure. This was considered as a measure of a user’s involvement in the network. For YouTube videos, the number of subscribers was used to determine the most influential users and videos in the network structure.

Frequencies of top words, the top words used and mentions of a ‘diet’ for MS were identified to gain insights into discourse within the network. The metric of number of followers for the top ten users in the networks, in addition to their favourite and repost count, were obtained for both platforms. The top twenty videos identified in the YouTube search were sorted by view count and viewed in full to categorise their source and potential endorsement of an MS-focused diet(s). For our study, endorsement referred to the promotion or support of a predefined diet that targeted plwMS.

#### Quality of information

We used the QUEST (Quality Evaluation Scoring Tool) to analyse the quality of the YouTube social media information. The QUEST tool is a seven-item validated questionnaire to assess the quality of online health information based on authorship, attribution, conflict of interest, currency, complementarity, and tone. A QUEST score was calculated by the primary researcher [blinded for review] for all videos, with a 10 % random sample analysed by a secondary researcher for quality assurance. A weighted score was created for an overall score of between 0 and 28. A higher score indicating better quality video content^(30)^.

#### Statistical analysis

Quantitative social media metrics and QUEST data were exported to SPSS software (v25, IBM). Frequencies were obtained for categorical variables and normality tests using the Shapiro–Wilks test. Non-parametric data are presented as median and IQR. Chi-square/Fisher’s exact and Mann–Whitney tests were performed to examine factors associated with the endorsement of an MS diet and the information source. Correlations based on the normality of the data were calculated between the number of likes, dislikes, comments, views and subscribers and QUEST scores and for the NodeXL Twitter/X data for favourite and repost counts.

A logistic regression model examined the relationship between videos endorsing an MS-specific diet (dependent variable) and several predictor variables: purchase encouragement, academic references, verified source, QUEST score and number of subscribers. The assumptions of logistic regression, including linearity and multicollinearity, were tested and satisfied. Outliers were considered on an individual basis. Due to the exploratory nature of our study, no adjustments were made to the models.

#### YouTube content analysis

The information source was determined by the most prominent voice in the video. For multiple voices, videos were classified by the source whose voice represented more than 50 % or the largest proportion of the video content. Based on a study by Basch et al.^(31)^, the information source was classified into five categories: healthcare professional, layperson, non-government organisation, news clip and other with healthcare professional further categorised as: doctor (including both general practitioner (GP/physician and specialists), dietitian, personal trainer, physiotherapist, naturopath, nutritionist and hospital. The term physician has been used in this study as all instances of the term doctor referred to a person who was medically trained. Dichotomised categories of nutrition trained and not nutrition trained were used for analysis of the healthcare professional category. To be classified as nutrition trained, the speaker had completed tertiary training in nutrition and/or dietetics through a known training programme, for example, accredited dietetic programme, due to the high variability of nutrition content within health degrees. Nutrition needed to be recognised as comprising most of the course content. Food science training, for example, was not eligible.

The top ten videos, identified through the NodeXL closeness centrality (reachability) metrics, were transcribed verbatim. NVivo software (version 12, 2018) was used to manage the content analysis using the framework approach^(32)^. This involves coding transcripts line by line and refining the themes^(33)^. Researcher biases were journaled prior to the analyses. Scientific claims made within the videos that related to diet and MS were compared with scientific evidence to aid identification of misinformation^(34)^. The claims made were verified by a senior dietitian specialising in MS research. Consensus on an appropriate sample size was not available; hence, data analysis ceased when repetition of topics and concepts was evident^(35)^.

### Data synthesis

To detect potential misinformation, the validated model by Kumar et al. for online social networks was adapted^(36)^. The model is based on the principles of cognitive psychology and considers misinformation through three domains: (1) credibility of the source; (2) consistency and coherency of the message; and (3) general acceptability. The model was validated for use with Twitter/X and guided our classification of misinformation using both Twitter/X and YouTube data.

To examine the source credibility, the source of each post/video was classified into three categories (coherency, consistency, and acceptability)^(36)^. Coherency and consistency were assessed from the diets identified through NodeXL and YouTube analyses, scientific claims identified during content analyses and QUEST scores. Acceptability of posts were assessed using the number of likes/dislikes/reposts in conjunction with PageRank (influence) metrics from NodeXL. Acceptability of the videos was assessed by user interaction (number of views, comments, likes and dislikes).

## Results

### Social network analysis

The ‘multiple sclerosis nutrition’ keyword search on Twitter/X revealed zero vertices (users), while the word network of ‘multiple sclerosis diet’ revealed thirty users; however, the top ten users were unrelated to the topic. Eight out of ten YouTube users had direct Twitter/X handles which were included in the user network. For this, one YouTube account for an organisation did not exist on Twitter/X, though a representative of the organisation did and was used in its place. The most followed users in the Twitter/X user network are shown in online supplementary material, Supplemental Table 2. Words from the text analysis occurred 309 times and were used most by an account representing 72·8 % of the user network. The most common diets mentioned on Twitter/X were Wahls (57 times), paleo (15 times), ketogenic (11 times), vegan (9 times) and intermittent fasting, Swank and McDougall (1 time each) (Table 2). The ‘favourite count’ was strongly correlated with ‘repost count’ (r = 0·83, *P* = 0·000).

**Table 2.** Diets mentioned in Twitter/X and YouTube networks

Diet keyword	YouTube network		Twitter/X network	
	Mentions in description (*n*)	Mentions in title (*n*)	Mentions in post (*n*)	Origin of post^*^
Vegan	433	7	9	Organisation 1 (*n* 9)
Wahls	383	0	57	Doctor 1 (*n* 56)
				Doctor 2 (*n* 1)
Ketogenic	248	4	11	Doctor 1 (*n* 11)
Intermittent fasting	179	6	1	Doctor 2
Paleo	127	4	15	Doctor 1 (*n* 15)
McDougall	107	0	1	Organisation 1
OMS	85	19	0	–
Swank	5	0	1	Doctor 1

OMS, overcoming multiple sclerosis.

* Names of organisations and individuals have been replaced with a pseudonym where the social media handle was identifiable.

**Figure 1.**
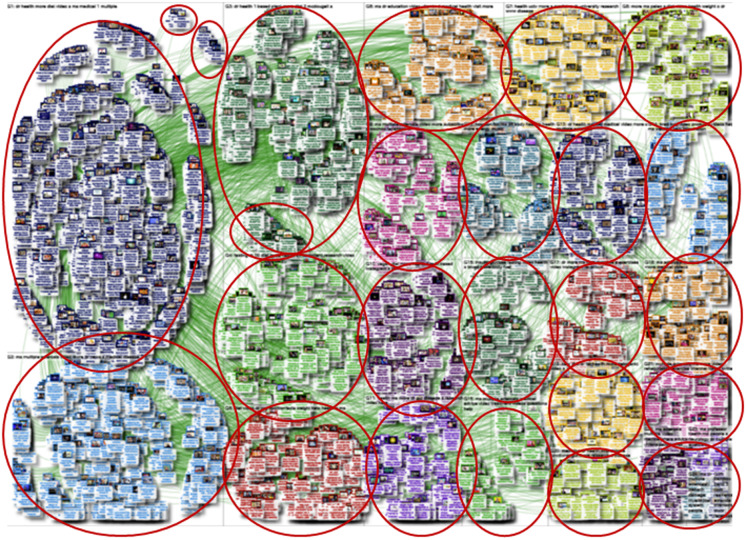
Clustering of users in social network structure for MS. MS, multiple sclerosis.

For YouTube, the search ‘multiple sclerosis diet’ revealed 100 users and 2902 edges (relationships between users), while ‘multiple sclerosis nutrition’ revealed 4096 users and 7094 relationships between users; hence, this search was used. The top five words were dr, health, ms, more and video. The SNA visualisation graph was ‘directed’, meaning that the relationships between users had a clear origin. The dominant network structure followed a shape referred to as a force-directed layout. This means the users repel each other, while the edges (relationships) pull the users together, creating a clustered network structure^(29)^ (Figure 1(b)). A total of twenty-five of users were identified using the cluster algorithm, and the top ten groups included 67·6 % of the relationships between users. Diets referred to in these groups included plant-based, McDougall (low fat and vegan), intermittent fasting, ketogenic, paleo and vegan (see online supplementary material, Supplemental Table 2). The words vegan and Wahls (creator of a modified paleolithic diet) were most frequently occurred in the video descriptions, and the OMS (Overcoming MS) diet was most common in video titles (Table 2). These diets were also searched in the Twitter/X user network, with Wahls featured in the greatest number of posts followed by paleo and ketogenic. The source of the top twenty videos by closeness centrality (reachability) were organisation (*n* 7), layperson (*n* 5), doctor (*n* 3), TV show (*n* 3) and university (*n* 2). The most influential video as determined by the number of subscribers was one which endorsed the author of the Wahl’s diet. Author-related TEDx talks had the most views, likes and comments overall.

### YouTube content analysis

**Figure 2.**
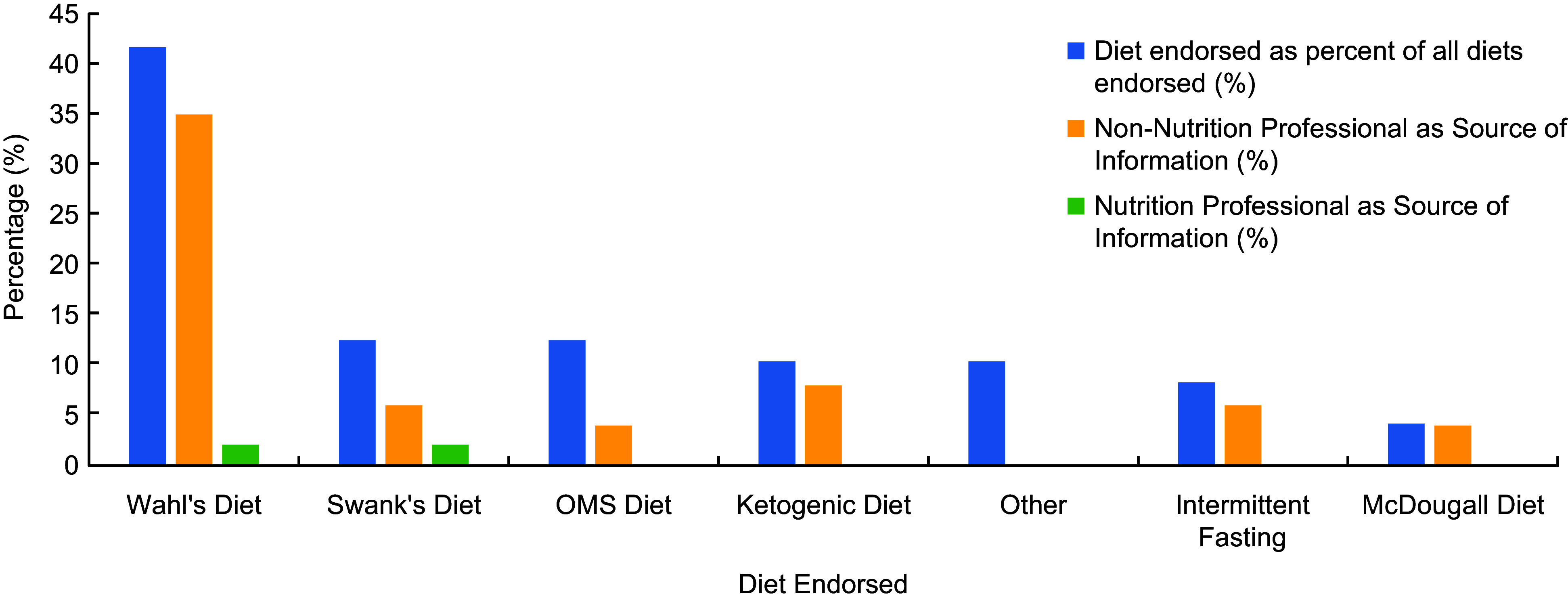
Diets endorsed by healthcare professionals within YouTube videos and sources of endorsement. Videos with source ‘Other’ (i.e. non-healthcare professional) were not included.

A total of 121 videos were eligible for inclusion (see online supplementary material, Supplemental Figure 1). Source categories included healthcare professionals (71·9 %), layperson (11·6 %), non-government organisation (10·7 %), news clip (5 %) and other (0·8 %). The types of healthcare professionals were doctor (76·7 %), dietitian (11·1 %), nutritionist (5·6 %), personal trainer (2·2 %) and physiotherapist, naturopath (1·1 %, each). An interview was the most common format (33·9 %), followed by educational style (24·8 %), webinar (10·7 %), presentation (8·3 %) and news segment (5 %). The USA was the most common country of origin of the videos (81 %), followed by Australia (9·9 %), England (4·1 %), Canada (2·5 %), Spain (0·8 %), India (0·8 %) and Wales (0·8 %). Only 17·4 % of users were YouTube-verified, meaning that the content belonged to an established creator or was an official channel of a brand or organisation. Most videos (69·4 %) discussed diet in relation to disease management, 1·7 % discussed diet in relation to disease prevention and 28·9 % discussed both prevention of onset and management of MS. The terms cure or recover/y in relation to diet and nutrition appeared ten times in five of the top ten videos. Most videos (71·9 %) did not cite scientific references to support the claims made. In 11·7 % of videos, viewers were encouraged to purchase a product or service related to the content, with a specific ‘MS diet’ endorsed in 39·7 % of videos. The Wahl’s diet was most common (41·7 %) and promoted by healthcare professionals who were not nutrition-trained. Non-nutrition-trained professionals endorsed all diets more than nutrition-trained professionals (Figure 2).

Continuous data were non-parametrically distributed due to highly influential videos. Sensitivity analyses removed two extreme outliers indicated by boxplot. Data differences were evident (see online supplementary material, Supplemental Tables 3–6); hence, outliers were excluded from all analyses. The median QUEST score (*n* 121) was 17/28 (IQR = 11–20), equivalent to 60·7 %. The median number of comments was 3 (IQR = 1–41), and the median number of subscribers was 7090 (IQR = 1410–89 000). The total QUEST score was negatively correlated with all social media metrics examined (number of views (R = –0·248), comments (R = –0·306), likes (R = –0·301) and dislikes (R = –0·271)). All correlations were weak to moderate with the strongest correlation between conflict of interest and the number of comments for a video (R = –0·409, *P* = 0·000). Analyses performed with elements of QUEST revealed conflict of interest (R = –0·409 to –0·270) and tone (R = –0·343 to –0·219) as negatively correlated with all metrics, while attribution was only negatively correlated with the number of comments (R = –0·042) (Table 3).

**Table 3.** Correlations between Quality Evaluation Scoring Tool (QUEST) scores and Twitter/X metrics

QUEST scoring element^†^	No. of views^‡^	No. of comments	No. of likes	No. of dislikes
Overall score (median score = 17/28)	−0·248	−0·306	−0·301	−0·271
	*P* = 0·007*	*P* = 0·001*	*P* = 0·001*	*P* = 0·004*
Authorship (median score = 2/2)	−0·138	−0·107	−0·75	−0·131
	*P* = 0·134	*P* = 0·270	*P* = 0·429	*P* = 0·165
Attribution (median score = 3/11)	−0·042	0·035	0·15	0·001
	*P* = 0·648	*P* = 0·717	*P* = 0·876	*P* = 0·993
Conflict of interest (median score = 6/6)	−0·270	−0·409	−0·399	−0·331
	*P* = 0·003*	*P* = 0·000*	*P* = 0·000*	*P* = 0·000*
Currency (median score = 2/2)	−0·098	0·075	0·042	−0·002
	*P* = 0·291	*P* = 0·438	*P* = 0·656	*P* = 0·981
Complementarity (Med. = 1/1)	N/A	N/A	N/A	N/A
Tone (median score = 3/6)	−0·219	−0·343	−0·311	−0·275
	*P* = 0·016*	*P* = 0·000*	*P* = 0·001*	*P* = 0·003*

Spearman’s correlation, *indicating Pearson’s correlation, *P* < 0·05.

^†^Scored using QUEST^(30)^. Authorship – clear mention of author’s name and qualification, Attribution – use of scientific evidence, appropriate references and quality of studies, Conflict of Interest – level of bias of information (i.e. does it endorse a product/intervention designed to prevent/treat a condition within the article), Currency – date of article (dated within last 5 years = highest score), Complementarity – support of the patient–physician relationship, Tone – use of balanced/cautious claims which include statements regarding limitations and/or contrasting findings, or fully supported claims which use strong vocabulary including non-conditional verb tenses (‘can’, ‘will’).

^‡^Each viewing occasion as independent.

**Figure 3.**
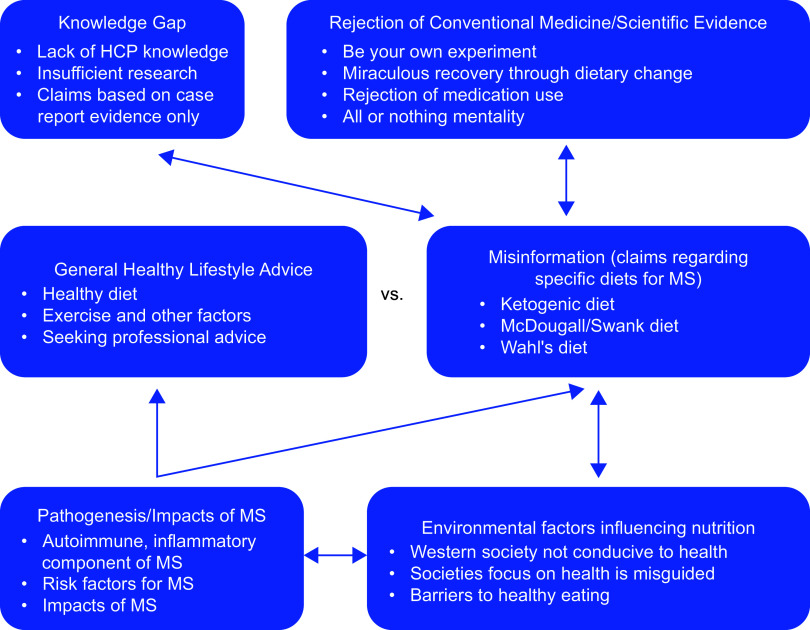
Relationships between themes and subthemes in YouTube videos showing relevant bidirectional relationships. HCP, healthcare professionals, MS, multiple sclerosis.

The content analysis identified conflicting nutrition advice for the management of MS. When videos were focused on a certain diet, this was often the sole topic. Subthemes and their relationships are presented in Figure 3 and explored below alongside exemplar quotes (see online supplementary material, Supplemental Table 8).

#### General healthy lifestyle advice

Healthy diet recommendations were aligned with dietary guidelines. Videos encouraged avoidance of excessive saturated fat aligned with the dietary guidelines. The benefits of exercise and other healthy lifestyle factors were also highlighted. Some videos recommended to seeking professional advice.

#### Misinformation (claims regarding diets for multiple sclerosis)

Many videos contained diet-specific claims that directly assist with MS-related outcomes, categorised as misinformation. The ketogenic diet was claimed to positively affect the brain. Although there is growing evidence for the ketogenic diet for MS^(37–40)^, claims within the videos used vocabulary in full support of the diet without acknowledging the extent of the evidence and related risks. The McDougall/Swank diets were also claimed to improve MS-related outcomes and assist in prevention. The Wahl’s diet further claimed to restore function and reverse MS. This diet encouraged daily consumption of nine cups of vegetables, including three cups rich in sulphur claiming to eliminate toxins.

#### Rejection of conventional medicine/scientific evidence

Videos encouraged viewers to be their own experiment, and claims were made that dietary change can lead to a recovery from MS with food classified as *‘medicine’*. There was a general rejection of medication use, and lifestyle change was considered superior to medication use in many videos. Some videos regarding self-management of MS claimed an all or nothing mentality with claims contradictory to the scientific evidence.

#### Health professional knowledge gap

Videos discussed a general lack of healthcare professional knowledge of nutrition management of disease, but also how dietitians may not have sufficient knowledge of MS. Insufficient research was also identified. Many animal studies were referred to in the videos, indicating a lack of human studies. Many claims were based on case report evidence^(41)^.

#### Environmental factors impacting nutrition

Many videos raised concerns about Western society being conducive to health, linking dietary patterns to disease. Videos also discussed how societal focus on health is misguided and focused on specific nutrients and/or supplements. Other barriers to healthy eating were identified, including familial influences.

#### Pathogenesis and impacts of multiple sclerosis

The autoimmune, inflammatory component of MS was frequently considered. Factors which exacerbate inflammation were discussed, including diet, exercise and stress. Risk factors for MS were addressed including cholesterol levels, gut dysbiosis and overweight/obesity. Despite the recognition of such factors, videos highlighted that the exact aetiology of MS is unknown, likely related to both environmental and genetic factors. Videos further discussed that disease can still occur in those with a healthy lifestyle. Videos referred to the many impacts of MS, including it being a *‘debilitating’* and costly. Various symptoms of the disease were articulated, reinforcing the impact of the condition.

## Discussion

The findings of our study provide insights into nutrition information related to the management of MS contained within Twitter/X and YouTube social media platforms. It addresses information that clients living with a health condition may face as they search online for health information. Our findings raise awareness of the influence of user engagement in social media platforms even when the source of the information may not be evidence-based. To our knowledge, this is the first SNA in relation to a condition such as MS and one of few SNA for a chronic condition. A strong interaction between platform metrics and quality of the information was found, with many factors driving the dissemination of content. Below, we discuss our findings using the Kumar model for misinformation in online networks^(42)^.

### Source credibility

YouTube videos created by healthcare professionals had higher QUEST scores, indicating that the information was of a higher quality than that from other sources. Healthcare professionals were more likely to cite scientific references; however, our study also found 71·9 % of YouTube videos did not refer to any scientific references. In the Twitter/X network, we found the most common domain was a US government-facilitated website, suggesting high source credibility. However, other common domains included social media platforms and non-government websites, indicating variability in source credibility. This finding is consistent with another Australian study that systematically explored web-based nutrition information relating to MS, indicating 72 % of pages did not provide full authorship and author credential information^(43)^. While the prior study did not use an SNA tool, it did use similar keywords to obtain their data.

Despite healthcare professionals generally producing higher quality videos, our study revealed they often lacked nutrition knowledge for MS management. This may be explained by insufficient research or the reported poor nutrition knowledge of neurologists^(44)^, the primary healthcare professionals for plwMS. This paucity of nutrition knowledge is well documented in a systematic review, indicating that medical students are not supported to provide high-quality, effective nutrition care, despite nutrition being a central tenet to a health behaviour change^(45)^. Additionally, our Twitter/X network found that some physicians were particularly active in the dissemination of nutrition mis/disinformation for MS when linked to the marketing of their own products or MS diets.

This is significant to the MS community as source credibility has been documented as a relevant cue to decipher online misinformation, shown to modulate the validation of implausible information^(46)^. This supports the discrepancy-induced source comprehension (D-ISC) assumption, suggesting that audiences become more conscious of the information source when they encounter discrepancies within it^(47)^. Therefore, audiences may be less inclined to critically appraise information if it is provided by a reputable source, such as a physician. Additionally, our study exemplifies this gap in the quality of nutrition information demonstrated online, as physicians were the most likely to endorse an MS diet that is not supported by current evidence. However, our data were skewed by influential accounts (physicians also living with MS); hence, the data should be interpreted with caution. This reiterates that, despite their credibility, some physicians may be inadvertently propagating nutrition misinformation.

### Coherency and consistency

Examination of the most prevalent diet words in the YouTube and Twitter/X networks revealed the words vegan, Wahls, ketogenic, paleo and OMS. This indicates a lack of consistency as these diets have insufficient evidence to support their efficacy for MS. A limitation of these findings, however, is that the words were only present in the titles and descriptions of the YouTube videos; hence, endorsement of these diets could not be specifically determined. A further limitation is that each mention of a diet within video titles and descriptions, and posts, was included in the total count; therefore, multiple mentions may have skewed the data. Equally, the view count of videos considers each viewing occasion as independent. It was not possible to determine if an individual viewed the same video multiple times. Regardless, these findings are supported by a scoping review of web-based recommendations for MS, which identified the paleo and Wahl’s diet, OMS and related dietary patterns as key recommendations for MS found online^(43)^. This contrasts with recommendations which encourage plwMS to follow the dietary guidelines of their country to ensure a balance of nutrients in the absence of more specific advice for MS. While many diets referred to online include elements of balance and dietary guidelines, many are restrictive or recommend avoidance of whole food groups or food types which may result in nutrient risk if not carefully monitored.

YouTube content analysis reiterated the lack of coherency and consistency, with some videos encouraging general healthy lifestyle advice including diet and others adherence to particular ‘MS diets’. Specifically, ‘MS diets’ were endorsed in 39·7 % of videos with these videos more likely to encourage viewers to purchase a product or service. This is consistent with the Academy of Nutrition and Dietetics position on nutrition misinformation, which indicates that such misinformation is promoted for financial gain^(48)^. Additionally, the median QUEST score for video quality was 61 %, higher in videos that did not endorse a specific diet. Logistic regression further found the encouragement of viewers to purchase a product or service (OR = 0·043, 95 % CI (0·003, 0·564)) and QUEST score (OR = 0·735, 95 % CI (0·636, 0·849)) as predictors for the endorsement of a specific diet, indicating financial motive and poor-quality information as potential influences of misinformation in our sample. The regression models also suggested no evidence of an effect related to the number of subscribers when a YouTube video endorsed a specific MS diet, as might have been expected (see online supplementary material, Supplemental Table 7).

### General acceptability

User interaction was negatively correlated with QUEST score, indicating engagement was higher in lower quality videos, and the reverse. When investigating elements of the QUEST tool, conflict of interest was negatively correlated with user interaction indicating higher engagement with biased information. This suggests that general acceptability may be greater in videos of lesser quality potentially because they include less scientific evidence which may confuse viewers, have the potential for data manipulation to support beliefs and utilise persuasive vocabulary^(49)^. Similarly, videos with lower scores for tone indicated greater bias and more user interaction, reiterating the above. Similarly, for Twitter/X networks, those who favourited more posts also reposted more posts, suggesting that they were more active in spreading information^(20)^. This indicates that more active sources may encourage the acceptance of misinformation, as acceptability increases with repeated exposure to an item^(36)^. However, the user with the most reposts made up 72·8 % of our network; hence, it cannot be determined if this user was spreading the most information as our results were skewed.

Demographic factors such as age, education level and income have also been negatively associated with the acceptance of health misinformation^(50)^. Findings from a systematic review indicate that information is more likely to be accepted if it is consistent with the user’s opinions and experiences^(51)^, referred to as confirmation bias. These factors were not examined in our study due to privacy and the unavailability of such data online, though they form an area of interest for future research.

Our study also found that the number of subscribers was significantly higher in videos which endorsed a diet for MS, suggesting higher user exposure to this information. Videos which endorsed a diet were also more likely to be YouTube-verified, reiterating their greater influence on viewers and suggesting a higher degree of acceptability of source. This could be explained by a lack of critical appraisal skills of audiences to decipher nutrition misinformation, consistent with previous studies^(50)^. The findings should be interpreted with caution as subscribing to a channel, and it being YouTube-verified does not necessarily predict an individual’s acceptance of all information that the channel is providing.

### Limitations

Due to the nature of SNA, the type of information obtained could not be limited to MS risk or management; however, this can also be considered as a strength as it facilitated the collection of unprecedented data and provides general insight into nutrition posts on social media related to MS. The creation of a user network for our study increased the available data, but we acknowledge that this may also increase the amount of unrelated content when not carefully managed. As our data were user-generated, it should be noted that it represents only individuals who willingly offered their opinions. This may influence the amount and/or type of information that was examined and should be interpreted with caution as those who do not share their options online may differ. In addition, demographic data cannot be obtained from social media data as many users give few or no explicit, self-reported characteristics in their user profiles^(52)^. This meant an analysis of differential patterns in attitudes and reporting behaviours is not possible. Similarly, the data obtained were in the English language only which may limit the perspective that the data are being viewed from as well as the lens (user voice) that the data is being shared from. The SNA data in our study were only obtained from two platforms over a 1-week period of time which offers exploratory insights into a niche area of health. While a longer period of data capture may reveal additional insights, it is expected that regular ‘voices’ of these social media platforms would have been captured during our data capture period due to the increased frequency of social media use globally. Further, as our data were collected in 2021, during the COVID-19 pandemic, it is likely that users were more aware of self-management options for their condition due to the restrictions that occurred for many health professions. This period also aligns with an increased engagement in online communications which may not be reflective of practices post-pandemic. The use of user networks in our methods may also have excluded less influential users who discuss diet/MS and prevented the attainment of the importance of a user for information spread within a given network. This can be useful for assessing changes to the way information spreads over time. SNA, generally, is also unable to capture representative outcomes due to the complexity and ever-changing nature of the data.

Correlation analyses comparing user interaction with QUEST scores were weak or moderate (r < 0·5) due to the small sample size and should be interpreted with caution. Similarly, the regression models while demonstrating some significant outcomes should also be considered with caution. While nutrition continues to be a regular topic of interest in the MS community, it is not expected that the amount of data captured would change post-COVID, though the amount of data for a different topic such as exercise may change. The content analysis was limited to a team of nutrition-trained researchers who may have influenced the interpretation of the YouTube content in comparison to a non-nutrition-trained researcher. Finally, YouTube is only one social media platform and cannot be considered representative of all social media platforms. Future studies may consider content analysis of other platforms such as Facebook or Instagram, each utilising different forms of online communication.

### Conclusion

This study highlighted the presence of potential nutrition misinformation within online social networks of YouTube and Twitter/X users. We also highlighted the variable quality of targeted YouTube video content. Our study found that users with large online followings may inadvertently disseminate potential misinformation and audiences should be encouraged to critically appraise the claims as they might for other sources. While influential accounts skewed our data, suggesting a need for further research, user engagement was found to be higher for biased and lower quality videos. This may suggest a lack of critical appraisal skills or awareness by the audience. In conclusion, potential nutrition misinformation relating to MS can be exposed using social media networks. Audiences should be cautioned about the influence of conflicts of interest and credibility of users with large online followings. Health professionals should be aware of the information-seeking behaviours in their clients and educate them about evidence-based findings.

## Supporting information

Probst et al. supplementary material Probst et al. supplementary material

## Supplementary material

For supplementary material accompanying this paper visit https://doi.org/10.1017/S1368980025100943
