# Supplementary material for: Examination of social media nutrition information related to multiple sclerosis: a cross-sectional social network analysis
Source: Public Health Nutr. 2025 Sep 18;28(1):e166. doi: 10.1017/S1368980025100943 (PMC12722084; doi:10.1017/S1368980025100943)
Supplement: Probst et al. supplementary material [file S1368980025100943sup001.docx]

SUPPLEMENTARY MATERIAL

Videos obtained from “multiple sclerosis and nutrition” search

n = 113

Videos obtained from “multiple sclerosis and diet” search

n = 100

Identification

Videos after irrelevant records excluded

n = 86

Videos after irrelevant records excluded

n = 98

Eligibility

Screening

Total videos from both searches

n = 184

Included

Total videos included after duplicates removed

n = 121

**Figure 1. Selection process for eligibility of the YouTube videos included in the content analysis**

Table 1. Twitter/X user network showing number of account followers/following and sentiment analysis list by user

| **User pseudonym^a^** | **No. of followers (n)** | **No. of accounts following, n(order #)** | **No. of mentions of “nutrition” or “diet”^b^, n(%)** |
| --- | --- | --- | --- |
| Person 1 | 3899084 | 716 (#7) | 2 (0.6%) |
| Organisation 1 | 348140 | 407 (#8) | 12 (4.2%) |
| Person 2 | 113032 | 734 (#6) | 1 (0.3%) |
| Person 3 | 31476 | 960 (#5) | 225 (72.8%) |
| Organisation 2 | 15307 | 3413 (#1) | 15 (4.9%) |
| Organisation 3 | 8612 | 1679 (#2) | 6 (1.9%) |
| Person 4 | 7732 | 154 (#9) | 15 (4.9%) |
| Organisation 4 | 2488 | 1142 (#3) | 23 (7.4%) |
| Organisation 5 | 934 | 1115 (#4) | 7 (2.3%) |
| Person 5 | 773 | 18 (#10) | 2 (0.6%) |

^a^ Names of organisations and individuals have been replaced with a pseudonym where the social media handle was identifiable.

^b^ NodeXL software, sentiment analysis

Table 2. Top 10 Groups identified using a cluster algorithm in the YouTube search

| Group | No. of videos | Key words |
| --- | --- | --- |
| 1 | 820 | dr health more diet video ms medical multiple |
| 2 | 406 | ms multiple sclerosis video more dr news medical disease |
| 3 | 374 | dr health **based** **plant** more diet **mcdougall** |
| 4 | 230 | **fasting** dr diet more health **intermittent** research video |
| 5 | 185 | diet video dr org nutritionfacts weight **keto** health ms |
| 6 | 177 | ms dr education video vitamin d medical health visit more |
| 7 | 147 | health uctv more medicine dr university research www disease |
| 8 | 146 | more ms **paleo** diet video health weight dr |
| 9 | 145 | ms multiple sclerosis video more subscribe woohoo facebook dr |
| 10 | 140 | **vegan** diet more health tedx based instagram talk |

Diet related terms indicated in **bold.**

**Table 3: Specificity test of descriptive statistics related to the eligible You Tube videos**

|  | Including outliers  (Median (IQR)) | Excluding outliers  (Median (IQR)) |
| --- | --- | --- |
| *Year posted* | 2018 (2016-2019) | 2018 (2016-2019) |
| *No. of views* | 2761 (597-16461) | 2621 (586-10840) |
| *No. of likes* | 50.5 (6-339) | 39.5 (6-306) |
| *No. of dislikes* | 2 (0-8) | 1.5 (0-7.25) |
| *No. of comments* | 3.5 (1-45) | 3 (1-41) |
| *No. of subscribers* | 7240 (1410-115000) | 7090 (1410-89000) |
| *QUEST score* | 16 (10-20) | 17 (11-20) |
| *Authorship* | 2 | 2 |
| *Attribution* | 3 | 3 |
| *Conflict of interest* | 6 | 6 |
| *Currency* | 2 | 2 |
| *Complementarity* | 1 | 1 |
| *Tone* | 3 | 3 |

**Table 4: Correlations of elements of the Quality Evaluation Scoring Tool, QUEST with You Tube video metrics (including outliers)**

| QUEST element | No. of Views | No. of Comments | No. of Likes | No. of Dislikes |
| --- | --- | --- | --- | --- |
| Overall Score  *(median score = 16/28)* | -0.173 (p=0.058) | -0.246 (p=0.009)* | -0.159 (p=0.089) | -0.200 (p=0.032)* |
| Authorship  *(median score = 2/2)* | 0.024 (p=0.794) | 0.06 (p=0.950) | 0.033 (p=0.728) | 0.12 (p=0.897) |
| Attribution  *(median score = 3/11)* | -0.002 (p=0.987) | 0.008 (p=0.930) | -0.23 (p=0.810) | -0.011 (p=0.903) |
| Conflict of Interest *(median score = 6/6)* | -0.210, (p=0.021)* | -0.310 (p=0.001)* | -0.193 (p=0.038)* | -0.242 (p=0.009)* |
| Currency  *(median score = 2/2)* | -0.210, (p=0.021)* | -0.164 (p=0.085) | -0.172 (p=0.065) | -0.191 (p=0.040)* |
| Complementarity *(median score = 1/1)* | N/A | N/A | N/A | N/A |
| Tone  *(median score = 3/6)* | -0.145 (p=0.112) | -0.230 (p=0.015)* | 0.093 (p=0.319) | -0.152 (p=0.104) |

*Spearman’s correlation, * indicating Pearson’s correlation, p<0.05.*

**Table 5: Chi-square and Mann Whitney analyses related to the eligible You Tube videos (including outliers)**

|  |  | ***Information Source*** | | | | ***Endorsement of a diet for MS*** | | | |
| --- | --- | --- | --- | --- | --- | --- | --- | --- | --- |
|  |  | *HCP (n=90)* | *Other (n=31)* | *Total (n=121)* | *P-value* | *Yes (n=48)* | *No (n=73)* | *Total (n=121)* | *P-value* |
| **Endorses specific MS diet^a^** | Yes | 33 (68.8%) | 15 (31.2%) | 48 | 0.174 | N/A | N/A | N/A- | - |
|  | No | 57 | 16 | 73 |  |  |  |  |  |
| **Encourages viewers to purchase something^a^** | Yes | 10 (71.4%) | 4 (28.6%) | 14 | 0.483 | 13 (92.9%) | 1 (7.1%) | 14 | 0.000 |
|  | No | 80 | 26 | 106 |  | 34 | 72 | 106 |  |
| **Cited academic references^a^** | Yes | 29 (85.3%) | 5 (14.7%) | 34 | 0.065 | 24 (70.6%) | 10 (29.4%) | 34 | 0.149 |
|  | No | 61 | 26 | 87 |  | 38 | 49 | 87 |  |
| **Source YouTube verified)^a^** | Yes | 11 (52.3%) | 10 (47.6%) | 21 | 0.011 | 17 (81.0%) | 4 (19.0%) | 21 | 0.000 |
|  | No | 79 | 21 | 100 |  | 4 | 69 | 73 |  |
| **Total QUEST score^b^** |  | 67.29 | 42.74 | - | 0.001 | 35.57 | 77.72 | - | 0.000 |
| **Source number of subscribers^b^** |  | 51.60 | 61.48 | - | 0.158 | 73.45 | 41.43 | - | 0.000 |

*^a^Chi-square/Fishers exact test, ^b^Mann Whitney test, p<0.05.*

*QUEST: Quality Evaluation Scoring Tool*

**Table 6: Logistic regression model analysing variables associated with the endorsement of a specific MS diet related to the eligible You Tube videos (n=107)**

| Variable | Odds Ratio | 95% CI | P-Value |
| --- | --- | --- | --- |
| Encourages viewers to purchase something | 0.043 | 0.003-0.564 | 0.017 |
| Cited academic references | 0.239 | 0.048-1.205 | 0.083 |
| Source YouTube verified | 0.154 | 0.021-1.149 | 0.068 |
| QUEST Score | 0.735 | 0.636-0.849 | 0.000 |
| No. of subscribers | 1.000 | 1.000-1.000 | 0.619 |

**Table 7: Content analysis themes of YouTube videos with exemplar quotes**

| **YouTube content analysis theme** | **Exemplar quotes** |
| --- | --- |
| *General healthy lifestyle advice* |  |
| Healthy eating | “high amounts of dietary fibre, fruits, vegetables” (V10, 57:57)  “fish is recommended at least twice a week” (V3, 4:44) |
| Exercise and other factors | “get stronger” (V7, 3:30)  “alleviate some of that pain in early onset fatigue” (V7, 3:36) |
| Seeking professional advice | “discuss doing under the guidance of your clinician” (V3, 9:11) |
| *Misinformation (claims regarding specific diets for MS)* |  |
| Ketogenic diet | “actually giv[e] your brain more power” (V8, 0:21)  “neuroprotection” (V8, 1:06)  “reduce the inflammation in the brain” (V8, 3:49) |
| McDougall/Swank diets | “MS is rare in parts of the world where people eat a healthy diet with little meat and dairy, and…common in parts of the world where people ate a lot of meat and dairy” (V2, 17:15) |
| Wahl’s diet | “restore the function of your brain” (V1, 20:36)  “actually reverse or greatly improve multiple sclerosis” (V1, 0:36)  “improve our ability to eliminate all of those toxins that…had likely stored in my fat and contributed to a decline” (V1, 24:02) |
| *Rejection of conventional medicine/scientific evidence* |  |
| Be your own experiment | “it doesn’t matter what the literature says, what matters is what’s working for you” (V5, 28:36) |
| Recovery due to dietary change |  |
| Food as medicine | “disease modifying drug” (V1, 52:02) |
| Rejection of medication | “the treatments are horrible and criminally expensive” (V2, 45:54)  “the best medicine from the best people in the country is not stopping my march towards a bed ridden demented life” (V5, 3:09) |
| All or nothing mentality | “you have to commit to doing the diet and lifestyle recommendations 100% for 100 days” (V1, 43:36) |
| *Knowledge gap* |  |
| Lacking knowledge of health care professional knowledge | “diet and nutrition has not been taught in medical school for many years.” (V2, 56:49)  “my dietitian friends, they can’t help me...so I’m back to online searching” (V5, 15:02) |
| Insufficient research | “there is a surprisingly low amount of strong nutritional research in multiple sclerosis” (V10, 56:28) |
| Case report evidence only | “Today 20 years after being diagnosed, she continues to thrive and help countless others with her diet and lifestyle therapies” (V5, 1:01)  “she became semi-vegetarian and started our diet in 2001 and stopped multiple sclerosis in 2002” (V2, 40:34) |
| *Environmental factors impacting nutrition* |  |
| Western society not conducive to health | “the fact is that these diseases have not really been important more than 70, 80, 90 years ago, and we do see this increase is usually associated with a modern lifestyle” (V10, 19:07) |
| Societies focus on health is misguided |  |
| Barriers to healthy eating | “I follow gluten free, dairy free 100%. My big struggle is when my grandkids come” (V1, 46:51) |
| *Pathogenesis and impacts of MS* |  |
| Exacerbating inflammation | “conventional standard American diet full of sugar” (V5, 26:56)  “being very sedentary” (V5, 27:03) |
| Impact of MS | “cost [that] is very high both to its society and the individual” (V1, 4:37)  “her symptoms left her unable to walk or continue doing her job” (V4, 0:05), “the fatigue was impacting every aspect of my life” (V1, 7:47)  “I was depressed. It was not a good time” (V1, 9:26) |
